# Differential Use of Pediatric Video Visits by a Diverse Population During the COVID-19 Pandemic: A Mixed-Methods Study

**DOI:** 10.3389/fped.2021.645236

**Published:** 2021-07-12

**Authors:** Jennifer L. Rosenthal, Christina O'Neal, April Sanders, Erik Fernandez y Garcia

**Affiliations:** Department of Pediatrics, University of California, Davis, Sacramento, CA, United States

**Keywords:** telemedicine, COVID-19, pediatrics, ambulatory care, health equity

## Abstract

**Objective:** To describe and explore pediatric ambulatory video visit use by patient characteristics during the coronavirus disease 2019 (COVID-19) pandemic.

**Methods:** We conducted an explanatory sequential mixed methods study with integration at the design and methods level. Phase 1 was a cross-sectional analysis of general and specialty pediatric ambulatory encounters to profile the use of video visits by patient characteristics. We performed descriptive analyses for each variable of interest and estimated a multivariable logistic regression model to analyze factors associated with the odds of having a video visit. Phase 2 was a qualitative exploration using semi-structured interviews with healthcare team members to understand the contextual factors influencing video visit usage. We used an interview guide to solicit information related to general perceptions about ambulatory video visits, reactions to the quantitative phase data, and strategies for optimizing equitable reach of video visits. Data were analyzed using a combination of deductive and inductive analysis.

**Results:** Among the 5,464 pediatric ambulatory encounters completed between March 11 and June 30, 2020, 2,127 were video visits. Patient factors associated with lower odds of having a video visit rather than an in-person visit included being Spanish-speaking (aOR 0.27, 95% CI 0.20–0.37) and other non-English-speaking (aOR 0.50, 95% CI 0.34–0.75) in comparison to English-speaking. Patients with public insurance also had a lower odds of having a video visit in comparison to privately insured patients (aOR 0.77, 95% CI 0.67–0.88). Qualitative interviews identified five solution-based themes: (1) Promoting video visits in a way that reaches all patient families; (2) Offering video visits to all patient families; (3) Mitigating digital literacy barriers; (4) Expanding health system resources to support families' specific needs; and (5) Engaging and empowering health system personnel to expand video visit access.

**Conclusion:** We identified differences in pediatric ambulatory video visit use by patient characteristics, with lower odds of video visit use among non-English-speaking and publicly insured patients. The mixed-methods approach allowed for the perspectives of our interview participants to contextualize the finding and lead to suggestions for improvement. Both our findings and the approach can be used by other health systems to ensure that all patients and families receive equal video visit access.

## Introduction

Telehealth is defined as the use of medical information exchanged via electronic communications to support and provide health care (1). Live encounters that involve real-time, synchronous, bi-directional audio and videoconferencing between patients and their health care providers (hereafter known as “video visits”) are a form of telehealth. Recognized benefits of video visits include mitigating healthcare access barriers related to geography, time, finances, and unique circumstances such as travel burdens for technology-dependent children (2–4). The American Academy of Pediatrics promotes telehealth as a strategy to increase continuity, efficiency, and quality in pediatric healthcare (5).

The coronavirus disease 2019 (COVID-19) pandemic has accelerated the adoption of video visit use for ambulatory patient care encounters (6–10), in great part to preserve personal protective equipment and minimize the transmission risk of infection to healthcare providers, patients, and families. In order to facilitate the adoption of video visits during this unprecedented time, changes in reimbursement, HIPAA (Health Insurance Portability and Accountability Act), and licensure regulations have been implemented (11–13).

Experts have raised concerns that the expanded use of healthcare-related technology during COVID-19 can exacerbate healthcare disparities for vulnerable populations despite the promise for improving healthcare outcomes overall (14–16). There has been limited but important work describing the aspects of video visits that may limit their use in specific populations including low digital health literacy, cultural preference for in-person visits, and limited access to reliable internet or technological devices (e.g., smartphones, tablets, computers) that are required to conduct a video visit (17). However, research is necessary to better understand the patterns of video visit use by patient characteristics during this important time in history. Our objective was therefore to describe and explore pediatric ambulatory video visit use by patient characteristics during COVID-19. The overarching question that guided this mixed methods study was: How does video visit use during COVID-19 for pediatric ambulatory encounters differ depending on patient characteristics, and what are the contextual factors that influence video visit usage?

## Materials and Methods

### Mixed Methods Integration

We conducted an explanatory sequential mixed methods study. This two-phase design began with a cross-sectional analysis of pediatric ambulatory encounters using electronic health records to profile the use of video visits (i.e., quantitative phase). The second phase was a qualitative exploration using semi-structured interviews and a combination of inductive and deductive methods to generate contextualized understanding of video visit usage (i.e., qualitative phase).

**Figure 1:**
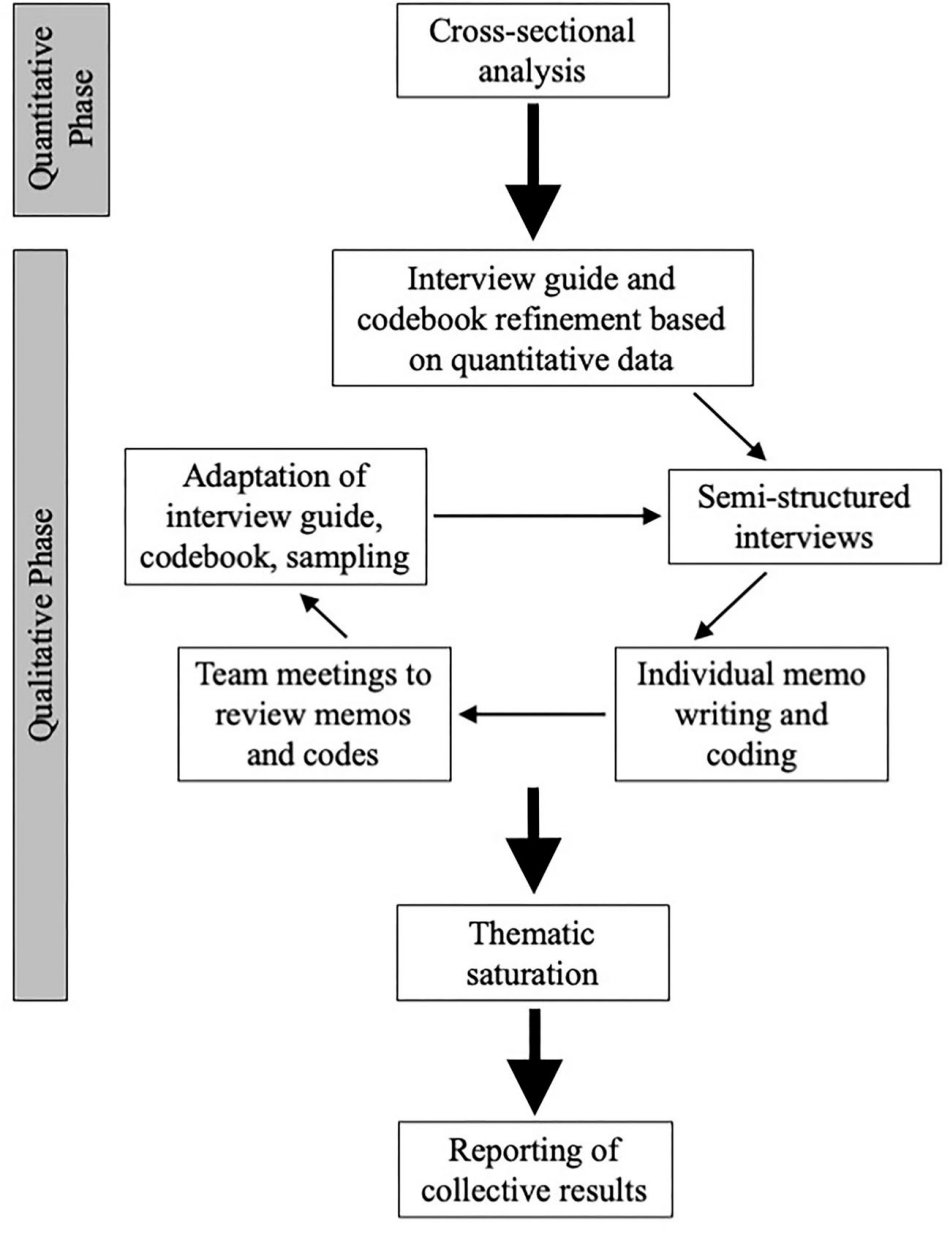
Study methodology.

In addition to implementing integration at the design level, we integrated at the methods level through connecting (18), whereby the results from the quantitative phase informed the sampling criteria regarding the types of providers recruited for the qualitative phase. Furthermore, we implemented integration through building; (18) we used the quantitative data to refine our interview guide and develop our deductive codes. Figure 1 shows an overview of the study methodology.

### Setting

This study took place at a quaternary care academic health system with an integrated primary and specialty ambulatory clinic network in Northern California. This medical center is the referral center for children across a 33-county region covering 65,000 square miles and serving over 1 million children. More than 120 physicians provide ambulatory care to our pediatric patients and families.

Our health system began offering pediatric ambulatory video visits in March 2019. Prior to COVID-19, the use of pediatric ambulatory video visits represented 1% of all pediatric ambulatory visits. We used the telehealth platform Epic MyChart (Epic Systems, Verona, WI) to conduct video visits. Parent or guardian (referred to as “parents” hereafter) requirements to conduct a video visit included establishing a MyChart account for the child and having a video-enabled smart device with WiFi or cellular access. The clinician requirements to conduct a video visit included having an iOS smart device with WiFi or cellular access. Clinicians could not use a desktop computer nor Android device, as these equipment types were not supported by our telehealth platform.

The quantitative phase of this study began during the initiation of a statewide COVID-19 shelter-in-place order. Initially, the general pediatric clinic tried to restrict in-person visits to children 2 years and younger. This policy was lifted after 2 months and did not occur in specialty clinics. General and specialty clinic physicians reviewed their scheduled patients to determine which appointments could convert to video; this decision was at the discretion of the physician. Two months into the study, safety protocols were implemented (e.g., universal masking with face shields, isolation rooms), and the pediatric clinics began scheduling new appointments as in-person visits again. Visitation restrictions permitted one parent to attend the in-person visits with the child.

### Quantitative Phase

#### Patient Population

For this cross-sectional analysis, we included all patients age 0–25 years who completed an ambulatory encounter with a Department of Pediatrics physician between March 11 and June 30, 2020. We included both in-person and video visits. All general and specialty ambulatory pediatric clinics located on the medical center campus were included; we excluded satellite and outreach clinics. Ambulatory encounters for procedures were excluded. We included patients up to age 25 years in order to include the young adults seen by pediatric physicians.

#### Data Source and Variables

We obtained patient-level data from the electronic health record. Patient characteristics included age, gender, race/ethnicity, language, insurance status, clinic type (surgical specialty, non-surgical specialty, general), and driving distance from residence to clinic (determined using the Google Cloud Platform's Distance Matrix API).

#### Analysis

We performed descriptive analyses for each variable of interest. For each categorical characteristic, we compared data between in-person visits vs. video visits using Pearson's chi-square tests. For each category, we calculated the proportion of video visits as a ratio of the number of video visit encounters (numerator) to the number of video visit plus in-person visit encounters (denominator). We estimated a multivariable logistic regression model to analyze factors associated with the odds of having a video visit, including covariates from the univariate analyses.

### Qualitative Phase

#### Interview Participants and Data Collection

We initially used purposive sampling (19) to identify clinicians and staff working in roles that would provide unique insights into the contextual factors influencing the video visit usage patterns observed from our quantitative data. Initial recruitment targeted telehealth program staff, information technology (IT) staff, professional medical interpreters, and physicians. We subsequently purposively sampled marketing staff to further explore marketing-related topics that arose in the initial interviews. We additionally used snowball sampling (20) to identify other potential contributory roles including triage nurses, clinic nurses, clinic managers, and medical office service coordinators.

One-on-one interviews were conducted from August through November 2020. Participants were recruited via e-mail. Eligible participants were aged 18 years and older and English-speaking. We collected demographic information during the interviews, including age, gender, race, ethnicity, clinical role, and years' experience. All interviews were conducted via videoconference and audio recorded and transcribed. Interviewers used an interview guide to structure the interview. The guide included questions to solicit information related to the following three topics: (1) general experiences with and perceptions about ambulatory video visits, (2) reactions to the video visits usage data from the quantitative phase, and (3) strategies for optimizing equitable reach of video visits. Interviewers maintained field notes with contextual observations. Each participant provided verbal informed consent and received a $50 gift card. Interviews were conducted until thematic saturation was reached.

#### Analysis

We used a combination of deductive and inductive analysis (21). Data were analyzed in an iterative process using a constant comparative approach (22, 23). We applied the quantitative phase results to develop an initial codebook of a priori codes related to the patterns identified in the data. Three team members (JR, AS, and EFG) independently conducted memo writing and coding of the initial five transcripts using the a priori codes while simultaneously identifying emergent codes. The team met virtually to discuss the relevance and definitions of the coding structure and new topics from inductive coding. Any team member who could not attend the meetings shared their memos and codes electronically; those memos and codes were reviewed at the team meetings and included in the discussions. We adapted the interview guide based on the initial codes. We then resumed independent memo writing and coding followed by team meetings to ensure consensus on application of codes, refine dimensions of existing codes, add new codes, develop categories, and identify theoretical direction. This iterative process was repeated with every 2–3 transcripts. We revisited prior transcripts as new codes were identified. We identified linkages and patterns between the codes, which became our analytic themes. Once data coalesced around similar themes across the participants' roles, we concluded that saturation of themes was met.

Data validation occurred through investigator triangulation (24). The qualitative analysis team consisted of a general pediatric attending (EFG), a telehealth medical director (JR), and a patient-centered care research coordinator with ambulatory clinical experience (AS). Two of the investigators (JR and EFG) had extensive qualitative research experience. An additional measure taken to enhance validity was the purposeful selection of the qualitative sample using the quantitative results to identify participants who could provide the best explanations (25). We used ATLAS.ti to organize and store coding and data analysis (26). This study was approved as exempt by the University of California Davis Institutional Review Board.

## Results

### Quantitative Phase

During the 16-week study period, there were 5,464 pediatric ambulatory encounters, of which 3,337 were in person and 2,127 were video visits. As shown in Table 1, Latinx/Hispanic patients were less likely to complete their ambulatory encounter by video (32.3%) than patients in other race/ethnicity categories (40.6–44.5%). For Spanish-speaking families, a greater percentage of them completed in-person visits (10.4%) than video visits (2.9%). Patients with private insurance were more likely to complete video visits (43.0%) than patients with public insurance (35.0%).

**Table 1:** Profile of patient-level characteristics for ambulatory video visits and in-person visits.

**Patient characteristics**	**Video visit**		**In-Person visit**		**Proportion as video visits, %**	***P***
	**(*****N =*** **2,127)**		**(*****N =*** **3,337)**			
Age, years, *n (%)*						<0.001
0–1	348	(16.4)	775	(23.2)	31.0	
2–5	418	(19.7)	705	(21.1)	37.2	
6–11	539	(25.3)	832	(24.9)	39.3	
12–18	752	(35.4)	952	(28.5)	44.1	
19+	70	(3.3)	73	(2.2)	49.0	
Gender, *n (%)*						0.015
Male	1,121	(52.7)	1,871	(56.1)	37.5	
Female	1,006	(47.3)	1,466	(43.9)	40.7	
Race & Ethnicity, *n (%)*						<0.001
Non-Hispanic White	1,030	(48.4)	1,283	(38.5)	44.5	
Latinx or Hispanic	466	(21.9)	976	(29.3)	32.3	
African American or Black	159	(7.5)	233	(7.0)	40.6	
Asian	212	(10.0)	280	(8.4)	43.1	
Pacific Islander	18	(0.9)	26	(0.8)	40.9	
American Indian or Alaska Native	13	(0.6)	8	(0.2)	61.9	
Other	41	(1.9)	178	(5.3)	18.7	
Missing	188	(8.8)	353	(10.6)	34.8	
Language, *n (%)*						<0.001
English	2,023	(95.1)	2,843	(85.2)	41.6	
Spanish	61	(2.9)	346	(10.4)	15.0	
Other	40	(1.9)	146	(4.4)	21.5	
Missing	3	(0.1)	2	(0.1)	60.0	
Insurance, *n (%)*						<0.001
Private	1,155	(54.3)	1,531	(45.9)	43.0	
Public	971	(45.6)	1,803	(54.0)	35.0	
Other	1	(0.1)	3	(0.1)	25.0	
Clinic Type, *n (%)*						<0.001
Non-Surgical Specialty	1,161	(54.6)	1,414	(42.4)	45.1	
Surgical Specialty	118	(5.6)	325	(9.7)	26.6	
General Pediatrics	848	(39.9)	1,598	(47.9)	34.7	
Distance, miles, *median (25–75% IQR)*	55.4	(27.2–155.7)	44.9	(21.4–112.6)		

*P-value determined using Pearson's chi-square test to compare data between in-person vs. video visits. IQR, inter-quartile range*.

#### Characteristics Associated With Having a Video Visit

In multivariable analysis, patient factors associated with higher odds of having a video visit rather than an in-person visit included age > 18 years, English-speaking, private insurance, and non-surgical specialty clinic (Table 2). Latinx/Hispanic patients had lower odds (based on the point estimate) of having a video visit in comparison to non-Latinx/Hispanic White patients (adjusted odds ratio [aOR] 0.86, 95% confidence interval [CI] 0.73–1.00). In comparison to English-speaking patients, Spanish-speaking patients (aOR 0.27, 95% CI 0.20–0.37) and other non-English-speaking patients (aOR 0.50, 95% CI 0.34–0.75) had lower odds of having a video visit. Patients with public insurance had lower odds of having a video visit in comparison to patients with private insurance (aOR 0.77, 95% CI 0.67–0.88). Regarding distance from the patient's home to the clinic, for every 100-mile increase in distance, the adjusted odds of having a video visit increased 1.25-fold (95% CI 1.19–1.32).

**Table 2:** Patient characteristics associated with having a video visit rather than an in-person visit.

	**Unadjusted OR, 95% CI**	**Adjusted OR, 95% CI**
Age, years		
0–1	0.47, 0.33–0.67	0.56, 0.38–0.83
2–5	0.62, 0.44–0.88	0.78, 0.53–1.14
6–11	0.68, 0.48–0.95	0.77, 0.53–1.13
12–18	0.82, 0.59–1.16	0.89, 0.61–1.30
19+	Ref	Ref
Gender		
Male	Ref	Ref
Female	1.15, 1.03–1.28	1.08, 0.96–1.22
Race & Ethnicity		
Non-Hispanic White	Ref	Ref
Latinx or Hispanic	0.59, 0.52–0.68	0.86, 0.73–1.00
African American or Black	0.85, 0.68–1.06	1.02, 0.81–1.29
Asian	0.94, 0.77–1.15	1.21, 0.98–1.49
Pacific Islander	0.86, 0.47–1.58	0.94, 0.50–1.74
American Indian or Alaska Native	2.02, 0.84–4.90	2.11, 0.84–5.30
Other	0.29, 0.20–0.41	0.41, 0.29–0.59
Language		
English	Ref	Ref
Spanish	0.25, 0.19–0.33	0.27, 0.20–0.37
Other	0.39, 0.27–0.55	0.50, 0.34–0.75
Insurance		
Private	Ref	Ref
Public	0.71, 0.64–0.80	0.77, 0.67–0.88
Other	0.44, 0.05–4.25	[—]
Clinic Type		
Non-Surgical Specialty	Ref	Ref
Surgical Specialty	0.44, 0.35–0.55	0.40, 0.32–0.52
General Pediatrics	0.65, 0.58–0.72	0.67, 0.59–0.76
Distance, 100 miles	1.22, 1.16–1.28	1.25, 1.19–1.32

*Associations with pediatric ambulatory encounters conducted as a video visit were compared with in-person visits. The multivariable logistic regression model included the covariates that are listed in this table. OR, odds ratio; Ref, reference*.

### Qualitative Phase

We conducted sixteen ~30–45-min interviews with individuals representing the telehealth program (*n* = 3), IT (*n* = 2), professional medical interpreting (*n* = 1), physicians (*n* = 2), marketing (*n* = 1), triage nurses (*n* = 1), clinic nurses or managers (*n* = 2), and medical office service coordinators (*n* = 4). Characteristics of participants are provided in Table 3.

**Table 3:** Interview participant characteristics.

	**n (%)**
Age, years	
25–34	5 (31.2%)
35–44	6 (37.5%)
45–54	4 (25.0%)
55+	1 (6.2%)
Gender	
Male	2 (12.5%)
Female	14 (87.5%)
Race & Ethnicity	
Non-Hispanic White	9 (56.2%)
Latinx or Hispanic	1 (6.2%)
African American or Black	1 (6.2%)
Asian	2 (12.5%)
Pacific Islander	1 (6.2%)
American Indian or Alaska Native	2 (12.5%)
Role	
Telehealth program staff	3 (18.8%)
Information technology staff	2 (12.5%)
Professional medical interpreter	1 (6.2%)
Marketing staff	1 (6.2%)
Physician	2 (12.5%)
Triage nurse	1 (6.2%)
Clinic nurse or manager	2 (12.5%)
Medical office service coordinator	4 (25.0%)
Years in profession	
<5	3 (18.8%)
6–10	5 (31.2%)
11–20	7 (43.8%)
21+	1 (6.2%)

We identified five overarching analytic themes across the transcripts that pertained to the contextual factors that influence video visit usage. Major themes were developed to be solution-based and included: (1) Promoting video visits in a way that reaches all patient families; (2) Offering video visits to all patient families; (3) Mitigating digital literacy barriers; (4) Expanding health system resources to support families' specific needs; (5) Engaging and empowering health system personnel to expand video visit access. These themes are explored in more detail below with representative quotes in Table 4.

**Table 4:** Exemplary quotes organized by theme.

**Theme**	**Exemplary Quote**
Promoting video visits in a way that reaches all patient families	“Low income like Medi-Cal families who maybe just aren't aware of the services are not potentially health literate in a way that they would advocate on behalf of themselves… I would assume that those types of families were under-served by the [video visit] service line… If you don't even know that there's services available to you, then you're in a position where you're not even making that decision on your own behalf.”—Telehealth Program Staff “Privately insured patients, usually that means [their parents] have a job. So, that's where their insurance is coming from. So, they are probably a little more assimilated or acculturated to the environment… Our low-income populations and populations with little information, they may not know that any of this is available and that this is easy and that this is just as good as your normal visit in the clinic. So, more education through Medi-Cal? That would be good too.”—Professional Medical Interpreter “I will just say that I do not think I've had a video visit with a non-English-speaking family. And I know that we have families who are signed up for MyChart whose parents are primarily Spanish-speaking or non-English-speaking, and even those families that have a MyChart account for their child, I have not had video visits with them. And my guess would be that the information that we provide, as an institution, all the way from the website level down to the information we provide in clinic, in person, that would help a family learn about video visits and about MyChart and accessing all of the capabilities it has, that we do not convey that as effectively to our non-English-speaking families.”—Physician “We have a lot of opportunity within our public affairs and marketing team to partner much more closely with our equity and inclusion department… We have really, really gorgeous, beautiful marketing, but how far is it reaching? And is it targeted enough?”—Telehealth Program Staff “There was some media push, I think, and this is all with COVID… ‘Hey, everyone should still get care, and you can get care in these different ways.' But I don't know if that extended beyond things like local news or local radio. I don't know if they went on Spanish language programming to try to help get that word out. So, it's always one of those things where you wonder, are we only talking to one subset of the population? Are we just skipping over a whole group of folks who would probably benefit from this?”—Physician “There is also a cultural reason for that, that the relationship with the provider is so important, because to them video visit doesn't feel like a full in-person—it's not the same. It's not the same experience, not the same feeling that you have established a relationship with the provider and that you're getting everything out of the visit that you would normally get when you showed up in the clinic… I don't see a wider outreach effort in order to normalize this and make this an option that is equal in quality to an in-person visit.”—Professional Medical Interpreter “…It scares them. It's a technology issue, language issue, the app is in English. Then when they are in the clinic, they know that an interpreter will show up in one way or another, whether phone, video or in person they will be there. With video visits they don't know. Will there be language support? What is going to happen? Yeah. It's a little unsettling to them. A cultural issue comes into play also, that this is not a real doctor visit, it's just all on TV, right?”—Professional Medical Interpreter “It seems like [video visits are] something that could cost more money, and there should probably be some kind of thought and research as to how we promote that cost point, and how we communicate that cost point.”—Marketing
Offering video visits to all patient families	“We should have a systematic approach across the board… it's not selectively sharing information. It's sharing information by default. So every person that walks into a clinic or every person that calls our nurse line is asked the same exact questions… ‘Do you have access to the portal?' Right? It removes all possible bias… Certain biases are just going to play out. So the more you can automate it and the more than you can put it actually in the—in the hands of the patient, the better your uptake.”—Telehealth Program Staff “First, unfortunately, people were actually told that only English-speaking patients could do video visits because there was not a great way to get interpreters involved. They did change that, and then there were multiple iterations of having interpreters involved… And now they can be ideally, easily in part of the visit.”—Telehealth Program Staff “I've heard anecdotally from providers, which makes total sense… they want a successful encounter. They want to feel comfortable. They are so busy, so trying something new is stressful… They think, ‘Oh, gosh, if the interpreter's not there, or they don't understand me, of if the connection drops,' then I mean there are just so many things that can go wrong… Connection is dropping, or the provider or clinician not knowing what to do or how to unmute themselves, or all these sort of like technology stressful things that they have to think about… So just wanting it to be in their control—that makes total sense to me that it wouldn't be the most comfortable thing to try something new or add onto their plate with the non-English-speaking family.”—Telehealth Program Staff “Also, ‘Are you biological mom?' Because if you're foster, or a guardian, then that's another hurdle to for Video Visit. So, as we're talking, even before I say, ‘Video Visit,' because I don't want to offer it to a foster mom who doesn't have all the paperwork.”—MOSC “There were people—potentially significant number of people that were hesitant at failure of a video visit, and that just creates some bias, whether subconscious or not of saying, ‘I'm not even going to offer it to you because I don't think you're going to be able to handle it.”'—Telehealth Program Staff “People of course have biases in their mind of who's going to follow through with the video visit. So Dr. X doesn't get mad at the person that scheduled it. You know? There are just so many ways on both sides that people want it to go well. So, they're going to lean toward the family that's probably there early. And maybe looks a certain way. And maybe has a certain type of insurance.”—Telehealth Program Staff “It seems like commercial patients are just, they're more responsible with MyChart. And following up with stuff like that, than Medi-Cal… And sometimes I'll skip that step [of enrolling them in MyChart], and that tends to be more Medi-Cal patients than commercial patients.”—MOSC “Clinic staff and people who know these patients probably have a good idea or sense, or even maybe a little bit of bias, in their mind in terms of do they no-show a lot? Do they maybe not follow through with things?… I heard doctors saying they wanted their patients and themselves to be set up for success. So if there's a family that maybe doesn't have great technology, they've said that before. Maybe they don't have phones or emails. They've said that before. I don't think they're going to try something new out, or something stressful with that particular family.”—Telehealth Program Staff
Mitigating digital literacy barriers	“The majority of the patients I think that I've seen who have done video visits have been those with private insurance. Again, those with higher SES, more medically sophisticated, so not necessarily patients whose parents have graduate degrees or other things, but people who have navigated the system well.”—Physician “Our experience is that with populations who are limited English proficient, or deaf populations it was a learning curve when the pandemic started and the video visits became the new normal. The instructions on how to log on, how to set it up were difficult, cumbersome.”—Professional Medical Interpreter “If you have a parent who's really technical and they can get on and they know how to do that, it's a walk in the park for them. But then, we might have our older folks—we have a lot of grandparents raising their grandkids nowadays, you know? So, that's a big one for them. I've had a couple of families that just, no matter how many times we've walked through it or we've showed them, or we've talked to them, they just still sometimes struggle, and I think that just is age and their savviness of technology.”—Clinic Nurse/Manager “Either they don't have the right technology, meaning their phone isn't up to date, or they don't have the right thing, or they just get so frustrated because they don't understand that they just want to give up, which is totally fine. So, then we usually just call the clinics, let them know what's going on, and then they'll change it to a telephone appointment.”—IT “Everything is in English. To sign up all of the terms and conditions that you're accepting are in English. So unless they have someone with them, or on the phone translating, that's tough. Of course, I mean we have now video visit instructions translated into Spanish, and working on other languages. But you still need—the whole system isn't translated. So, buttons are in English when you press ‘Begin Visit,' so that's tough.”—Telehealth Program Staff “Because the app is only offered in English, that's probably the biggest driving factor. I mean, I can promote it in Russian, but frankly, that's a little misleading if we're not actually offering it in Russian. So, I wouldn't suggest that we publish materials in other languages unless the end result is going to also be offered in other languages… I wouldn't want someone to be encouraged to sign up and download something, and then get in there and realize, ‘Oh, this isn't what I thought.”'—Marketing “I know some of our families that I think definitely would benefit, but… I think it's unfair because of the language barrier. So, we're asking them to sign up for this things that's completely in English and it's like, that would be like them telling us to sign something, for me, I don't speak Chinese. So, it would be like, ‘Okay, all this is in Chinese, but sign up for it.' And you're looking at it like it's completely foreign, so I wish and hope that it would be in other languages, just to make it more user friendly for our people, our patients, our families.”—Clinic Nurse/Manager
Expanding health system resources to support families' specific needs	“We'll have to do a better way of identifying why those gaps are there, and trying to meet them. But I think updating your website to be more clear, and maybe use bullets instead of paragraphs to communicate. And making sure we're translating.”—Telehealth Program Staff “Having more extensive social work and care coordination that's culturally competent around specialty and primary care and really understanding who our populations are and targeting—targeting interventions that meet those people where they need to be met.”—Telehealth Program Staff “I'd still say that video visits where I use an interpreter are still in the minority of what I do, and I think a lot of it is just like, I think a lot of it goes to the amount of time our nurses will spend…. They will look at the clinic schedule and they will make it a point to contact the family… But it's very time consuming, and in the past few months, they haven't been able to do that as much, because now it's just super overwhelming.”—Physician “Parents usually would spend a good amount of time with the interpreter on the phone just trying to get them through the steps of getting into the video visit. So, very frustrating on all sides… It was so frustrating and they would just rather come into the clinic… So, some gave up and started coming in when the clinic's opened again.”—Professional Medical Interpreter “[With COVID] our workload has just gone through the roof. So, it's like we can't spend as much time with these patients to get them 100 percent comfortable if they're not already tech savvy or they're not comfortable resolving issues on the fly as they go… It's that balancing act of how do I make sure the patient's good and how do I make sure my team's good so we're meeting our daily numbers.”—IT “We need more [IT Help Desk] people. We are hiring more people, so I'm optimistic. Here's the thing. With hiring more people, even still, I mean, what we've been told to do, the quality is suffering… Yes, [the patients' families are] getting a call, but it's in one ear and out the other. I hope that maybe having more people will help that, but still it's so much volume. There's so many people that I don't know if they're going to be getting the experience that I would personally want them to have.”—IT “They'll say, ‘Well, the kid doesn't have a cell phone… Can we use mine?' It says no; it's gotta be the child's… [The teens] have to have their own email or cell phone, because it's the kid's medical record. Because they have to have full access to have a Video Visit to MyChart.”—MOSC “I have a patient yesterday who specifically said that they live in a rural setting and they do not have a very reliable high-speed internet connection.”—Physician “Big hurdles that I've heard from families are like, ‘I'd love to do a video visit, but the thing is, we only have one computer for the whole household. If the other sibling is using the computer for school, then I can't use it for this.' In some of our families that live in really rural areas, I have to admit, it was very educational for me. Internet access is not as ubiquitous as I would have imagined, and so that was a huge challenge.”—Physician
Engaging and empowering health system personnel to expand video visit access	“And then I think sort of calling out the data, too, so people know that this is happening. Like, ‘Did you know that we as a department only saw X percent of non-English-speaking patients through video visit?' I think without people sort of showing this disparity and this gap in care to people, then having that awareness will hopefully help people to change.”—Telehealth Program Staff “How do you turn this situation around? It takes the individualized approach, it takes patience, it takes working, putting resources into it. So, if we are looking for volume, then yes, they'll come back. They're back in clinics in high numbers. But that's not what we are looking at, right? That's not the goal. The goal is maybe triage and handle appointments that can be done through video, do them that way. But, for LEP [limited English proficiency] populations they were just sitting tight and waiting for once the clinics would open back up.”—Professional Medical Interpreter “I would love to have more metrics and data around who's using it, and then within that, what demographics are using it, so that we can better identify who isn't using it, and hopefully find ways to advance this program amongst that population group… I would want to see age, race, language spoken, household income, things like that.”—Marketing
	“We made the impossible happen. Going from 100 video visits a week to 5,000 a week for COVID. So we can do even better now. I think smaller groups, showing the data, showing research to people, and then like what you're doing. Asking them, the people who are on the ground and part of it every day, of what would make it easier for everyone, to open up the access.”—Telehealth Program Staff “As far as the staff, I don't think that they feel that they have a big, powerful voice… For instance, my phone bank, my MOSC who's answering the phone, she might not feel like, ‘Hey, who do I even go to let them know this issue?'… So, you know, having somebody at the higher level who can make that decision understand that this is a real issue at the patient level.”—Clinic Nurse/Manager “I think that we need more dedicated effort to specifically reaching out to those groups that are underrepresented when it comes to MyChart enrollment, and in addition, those groups that are underrepresented for having a video visit done… It would be great to hear from the families themselves about what the barriers are so that whatever outreach we provide can be most effective. So, rather than offering a solution that we think will work, finding what solutions will work for those families, probably not going to be one size fits all.”—Physician “I think that it would be in our best interest to partner with the Office for Health Equity very frequently… That overall plan [to address video visit equity issues] needs to be built… The health equity group and communications would all be good to have at the table when that plan is being put together.”—Marketing

*MOSC, medical office service coordinator; IT, information technology*.

#### Theme 1—Promoting Video Visits in a Way That Reaches All Patient Families

Multiple participants across different roles reported that the awareness of video visits was not necessarily known by all of our diverse groups of patient families. Specifically, participants shared that lower-income, publicly-insured, and non-English-speaking patients were frequently less aware of the video visits option. This lack of awareness was thought to be a contributing factor for the quantitative results regarding lower odds of video visit usage among non-English-speaking and publicly-insured patients. Participants stated that limited awareness existed in part due to marketing mostly being in English and via the medical center's website or social media platforms, which thus may not reach those populations noted in the quantitative data of having lower use.

Another aspect highlighted by participants was the need to address parents' skepticism about video visits. They perceived that parents of publicly-insured children sometimes misunderstood their insurance coverage and feared that a video visit was more costly than the in-person charges (or lack thereof) to which they were accustomed. Participants also mentioned that non-English-speaking parents did not always know that a professional medical interpreter was available for video visits. These factors were thought to drive certain parents to preferentially use in-person visits.

#### Theme 2—Offering Video Visits to All Patient Families

Many of the participants shared that clinical providers and staff offered video visits less frequently to certain groups of patient families. Participants explained that inconsistent workflows and training on to whom to offer video visits resulted in this practice. In addition, almost every participant commented that navigating the video visit process was challenging. Therefore, any additional element that could make the process even more difficult—whether it be a real or perceived element—exacerbated the selective offering of video visits. Participants said that biases about a family influenced the perceptions on who was more or less likely to navigate the process. In particular, English-speaking parents and those profiled as “tech savvy” were often categorized as people who would be successful in navigating video visits. Additionally, video visits for patients in foster care required the foster parent to complete additional paperwork and this may lead to video visit failure. Thus, video visits were almost never offered to children in foster care. Some participants emphasized the need to standardize the video visit scheduling process in order to overcome the practice of selectively offering video visits to the patient families deemed most likely to successfully navigate the video visit process.

#### Theme 3—Mitigating Digital Literacy Barriers

The most recurring topic discussed by all participants was that the video visit platform, MyChart, was very “difficult” and “cumbersome” to navigate. The most challenging aspect of using MyChart in the context of setting up video visits was parents' creating the account. As a result, patients whose parents could not set-up MyChart were unable to conduct their ambulatory encounter as a video visit and defaulted to in-person visits. Furthermore, the MyChart platform was only available to our patient families in English, and assistance in navigating the process for non-English-speaking parents was variable. This language limitation was thought to be a primary driver of the language gap identified in the quantitative data. Numerous participants stated that a multi-language platform is a necessary investment that should be prioritized.

#### Theme 4—Expanding Health System Resources to Support Families' Specific Needs

The need to expand video visit resources to better support our patients and families was consistently expressed among all participants. First, the video visit written instructions were perceived to be overly complicated and not useful for those with low health and digital literacy. Additionally, the instructions were almost exclusively in English. Participants reported that some fliers were translated into Spanish but not into other languages. However, again, they emphasized that language appropriate instructions have limited effect when the MyChart platform was only in English.

Second, participants across various roles shared that they needed more clinical staff and IT personnel than was provided to assist patients and their families with the video visit process. In the setting of COVID, the exponential growth of video visits has surpassed their ability to address every patient family's needs. Participants stated that, for certain patients such as those who did not speak English, video visits required more time and effort from the health system in comparison to in-person visits. As a result, participants stated that “it's too much work” to do a video visit for certain patients. Additionally, video visits required extra steps on the family's end. Some parents who wanted to have a video visit for their child were left feeling frustrated and ultimately gave up despite assistance from the health system. Others could not have a video visit due to lack of equipment or insufficient internet access.

#### Theme 5—Engaging and Empowering Health System Personnel to Expand Video Visit Access

Some participants articulated the need to raise awareness about the differential uptake of video visits by certain groups of patient families. They explained that frontline providers and administrators were likely not aware of the differences in video visit access in certain populations. Very few of our participants responded that there were differences in uptake of video visits by groups of patients defined by insurance or language status when asked the open-ended question about their observations of any differences in video visit use depending on patient or family characteristics. However, upon being presented with the quantitative analyses of this current study, almost every participant said, “I'm not surprised.” They requested that these analyses be disseminated so that data transparency could influence individual- and systems-level changes. In addition to increasing awareness, participants recommended empowering all providers to advocate for their patients' needs and to provide providers with the necessary resources to best support their patient families.

## Discussion

This mixed methods study profiled pediatric ambulatory encounters during COVID-19 and identified that being non-English-speaking and having public insurance were independent risk factors for lower odds of having a video visit rather than an in-person visit. The subsequent qualitative exploration provided a contextualized understanding of video visit usage and identified various factors perceived to contribute to the video visit access inequities. Our solution-based themes identified that strategies to improve equitable access include expanding the reach of video visit promotions, standardizing the process of offering video visits, enhancing resources to support all patient families, and engaging all health system personnel to address inequities.

The data from our present study adds to the growing body of evidence suggesting that the expanded adoption of telehealth during COVID-19 may be taken up by some groups of patients more than others. Our findings, that insurance and language are predictors of video visit use, are consistent with previous non-pediatric research highlighting this differential use. Data from one study examining internal medicine primary care visits suggested decreased video visit access among patients who were publicly-insured, non-English-speaking, and non-Hispanic White (14). Furthermore, Spanish-speaking patients and those enrolled in Medicaid have been found to be less likely to have access to technology that enables video visits (27). Qualitative research highlights that medical providers report seeing fewer patients with limited English proficiency than usual in the setting of increased video visits during COVID-19; low digital literacy and English-only telehealth platform instructions are described as factors contributing to low video visit usage among this population (28). Regarding our finding that the 0–1-year-old age group had the lowest odds of video visit use, this differential use is likely explained by the relatively high proportion of visits in this age group that involve immunizations and thus require an in person visit.

The limited pediatric-specific research on video visits during COVID-19 has examined video visit utilization among single specialties and has mixed results. One study of pediatric neurology encounters found video visits to be less frequent among patients in racial or ethnic minority groups (29). Another study of pediatric otolaryngology encounters demonstrated no change in the proportion of Spanish-speaking and Medi-Cal patients seen by the clinic when they transitioned from in-person visits to exclusively video visits; however, Spanish-speaking families were more likely to require rescheduling of their video visits, which the authors used as a proxy measure for barriers to access (30). This context of exclusively offering video visits is one potential explanation for why this pediatric otolaryngology study had different results than our present study. Nevertheless, the authors similarly concluded that increased staff support is a necessary investment to provide a sustainable level of video visits to patient families with language barriers.

Findings from our current mixed-methods study were presented internally and activated immediate actions to begin addressing the identified differential uptake of video visits by certain groups (Table 5). First, a multidisciplinary quality improvement team convened to decrease the difference between English vs. non-English-speaking patients for the percentage of video visits among all ambulatory visits. The improvement project used the study analyses to inform their key driver diagram and initial tests of change. As part of this improvement project, real-time data on video visit usage by language will be disseminated to telehealth program leadership and clinics. Second, the IT help desk received training on how to use interpreter services when assisting non-English-speaking patients and families. The help desk also has a targeted patient outreach workqueue. They proactively call patient families when a video visit is scheduled to offer their assistance; non-English-speaking patient families are on that list. Third, the telehealth program leadership began investigating the potential use of the multi-lingual MyChart platform. Simultaneously, IT Education and interpreting services are working to simplify patient family-facing video visit materials in order to ensure that resources are not only language-appropriate but also effective for those with low health literacy. Fourth, we implemented a streamlined workflow whereby our video interpreting services platform, Martti (Cloudbreak Health, Columbus, OH), integrates into the video visit encounters. Providers can either schedule an interpreter ahead of time, or providers can invite the interpreter to join the video visit on demand. Fifth, the health system is hiring patient navigators to assist patient families with their additional needs in navigating the video visit process. We will apply continuous improvement processes to identify how to optimize this new resource. Finally, recognizing that similar or additional differences in video visit use may exist in the adult population, a team began applying our approach with pediatric data to the adult ambulatory video visit data. We believe that a similar approach to internal analysis of video visit use by patient characteristics, reporting, and action would be valuable to other health systems and is reflective of quality improvement best practices.

**Table 5:** Local actions taken to address identified factors contributing to differential use of video visits and the theme(s) each action targets.

**Action**	**Description**	**Theme alignment**
Quality improvement team	Multidisciplinary quality improvement team organized to decrease the difference between English vs. non-English-speaking patients for the percentage of video visits among all ambulatory visits.	(1) (2) (3) (4) (5)
IT help desk outreach	IT help desk has a targeted patient outreach workqueue (which includes non-English-speaking patient families); they proactively call these patient families when a video visit is scheduled to offer their assistance.	(2) (3) (4)
Language-appropriate materials for low-literacy audiences	IT Education and interpreting services are creating language-appropriate video visit materials designed for low health literacy patient families.	(1) (2) (3) (4)
Integration with video interpreting services	Video interpreting services platform now integrates into the video visit encounters; interpreters can be scheduled or invited to join the video visit on demand.	(4)
Patient navigators	The health system is hiring patient navigators to assist patient families with their additional needs in navigating the video visit process.	(2) (3) (4)

*IT, information technology*.

*(1) Promoting video visits in a way that reaches all patient families; (2) Offering video visits to all patient families; (3) Mitigating digital literacy barriers; (4) Expanding health system resources to support families' specific needs; (5) Engaging and empowering health system personnel to expand video visit access*.

As our process of disseminating our findings internally reflects best practices, the strategies identified by our study participants to improve video visit use by all of the patient families reflected in our study are consistent with strategies being promoted by national public agencies. The Centers for Disease Control and Prevention COVID-19 Response Health Equity Strategy emphasizes the importance of disseminating materials that are tailored to be culturally relevant and linguistically relevant for diverse groups (31). Similarly, a recent publication on disparities in telehealth access for vulnerable populations recommended key actions that are similar to the solution-based themes from our present study (14). These recommendations included identifying potential disparities in access (e.g., monitor data), mitigating digital literacy and resource barriers (e.g., educate and train patients in digital skills), removing health system-created barriers (e.g., offer video visits to every patients), and advocating for changes to support sustained and equitable access (e.g., expanded low-cost or free broadband).

The findings in our study were specific to our medical center and may reflect circumstances and contextual factors that were unique to our setting. Other medical centers might not identify the same patterns that we found in our study. Nevertheless, many of the findings are likely transferable to other pediatric ambulatory practices, and our study highlights the importance of conducting such investigations that explore potential disparities in video visit access and areas for improvement. Another limitation in our study was that the appropriateness of the type of visit was not assessed. We thus could not determine if a video visit vs. in-person visit was warranted for a particular encounter. The qualitative findings represent only the perceptions from our group of participants. However, we interviewed a diverse group with respect to their clinical roles and experiences. Interview participants shared their perceptions of patient and parent experiences; however, we did not include patients or parents in this study. Gathering data from the perspectives of patients and parents was beyond the scope of this present study but should be explored in future research. Addressing patient families' unique needs requires their input and perspectives. Importantly, future research that gathers data from patients and parents should ensure diversity in participants in order to understand how to best support diverse populations with telehealth services. Furthermore, participants who agreed to participate could also have extreme perceptions. Despite these limitations, this mixed methods study provided useful information to inform interventions to improve the pediatric video visit program and mitigate access inequities.

In conclusion, our profile of pediatric ambulatory video visit use by patient characteristics during COVID-19 identified differences, with lower odds of video visit use among non-English-speaking and publicly insured patients. Our mixed-methods approach allowed for the perspectives of our interview participants to contextualize the finding and lead to suggestions for improvement. We found that expanded reach of video visit promotions, standardized offering of video visits, enhanced resources to overcome digital literacy barriers, expanded resources to support all patient families, and engagement of providers to address inequities are potential strategies that may be incorporated to improve equal access to video visits among our diverse patient population. Therefore, both our findings and the approach to obtaining them present models for other health systems to ensure that all patients and families receive equal opportunity to reap the benefits of video visits, during the COVID-19 pandemic and thereafter.

## Data Availability Statement

The datasets presented in this article are not readily available because of patient anonymity. Requests to access the datasets should be directed to the corresponding author.

## Ethics Statement

The studies involving human participants were reviewed and approved by University of California Davis IRB. Written informed consent from the participants' legal guardian/next of kin was not required to participate in this study in accordance with the national legislation and the institutional requirements. Written informed consent was not obtained from the minor(s)' legal guardian/next of kin for the publication of any potentially identifiable images or data included in this article.

## Author Contributions

JR conceptualized and designed the study, conducted interviews, analyzed the data and interpreted the results, drafted the initial manuscript, and revised and approved the final manuscript as submitted. CO conceptualized and designed the study, conducted interviews, interpreted the results, and revised and approved the final manuscript as submitted. AS and EFG analyzed the data, interpreted the results, and revised and approved the final manuscript as submitted. All authors contributed to the article and approved the submitted version.

## Conflict of Interest

The authors declare that the research was conducted in the absence of any commercial or financial relationships that could be construed as a potential conflict of interest.
