# Supplementary material for: Differential Use of Pediatric Video Visits by a Diverse Population During the COVID-19 Pandemic: A Mixed-Methods Study
Source: Front Pediatr. 2021 Jul 12;9:645236. doi: 10.3389/fped.2021.645236 (PMC8311026; doi:10.3389/fped.2021.645236)
Supplement: Supplementary file 1 [file Data_Sheet_1.DOCX]

**Appendix A:** Qualitative Interview Guide

***General experiences with and perceptions about ambulatory video visits***

1. Tell me about your overall experience and thoughts about video visits for pediatric patients.
2. How does a patient typically get scheduled as a video visit as opposed to an in-person visit?
3. Can you think of a time that you think a patient would have benefited from a video visit but, for whatever reason, instead had an in-person visit? Tell me about it.
4. Video visit use rose rapidly with the onset of COVID-19. Based on your observations, have you noticed any differences in video visit use depending on patient or family characteristics?
   1. (If yes) Tell me about them. What do you think is driving those differences.

***Reactions to the quantitative video visit usage data***

1. Our pediatric data suggest that English-speaking patients have higher odds of having a video visit. What is your initial reaction to that finding?
   1. Why do you think that difference exists between English- and non-English-speaking patients?
   2. Can you think of a time that a non-English-speaking patient/family would have benefited from a video visit but instead had an in-person visit? Tell me about it.
2. Our pediatric data suggest that privately insured patients have higher odds of having a video visit. What is your initial reaction to that finding?
   1. Why do you think that difference exists between patients with private versus public insurance?
   2. Can you think of a time that insurance status influenced whether or not a patient had a video visit? Tell me about it.

***Strategies for optimizing equitable reach of video visits***

1. What do we need to do in order to make sure all patients have equal ability to complete a video visit?
2. Is there anything else you want to share with me regarding video visits?
